# A Study of Accelerometer and Gyroscope Measurements in Physical Life-Log Activities Detection Systems

**DOI:** 10.3390/s20226670

**Published:** 2020-11-21

**Authors:** Ahmad Jalal, Majid Ali Khan Quaid, Sheikh Badar ud din Tahir, Kibum Kim

**Affiliations:** 1 Department of Computer Science, Air University, Islamabad 44000, Pakistan; ahmadjalal@mail.au.edu.pk (A.J.); 171274@students.au.edu.pk (M.A.K.Q.); 181483@students.au.edu.pk (S.B.u.d.T.); 2 Department of Human-Computer Interaction, Hanyang University, Ansan 15588, Korea

**Keywords:** accelerometer, activity detection system, healthcare, inertial sensors, reweighted genetic algorithm

## Abstract

Nowadays, wearable technology can enhance physical human life-log routines by shifting goals from merely counting steps to tackling significant healthcare challenges. Such wearable technology modules have presented opportunities to acquire important information about human activities in real-life environments. The purpose of this paper is to report on recent developments and to project future advances regarding wearable sensor systems for the sustainable monitoring and recording of human life-logs. On the basis of this survey, we propose a model that is designed to retrieve better information during physical activities in indoor and outdoor environments in order to improve the quality of life and to reduce risks. This model uses a fusion of both statistical and non-statistical features for the recognition of different activity patterns using wearable inertial sensors, i.e., triaxial accelerometers, gyroscopes and magnetometers. These features include signal magnitude, positive/negative peaks and position direction to explore signal orientation changes, position differentiation, temporal variation and optimal changes among coordinates. These features are processed by a genetic algorithm for the selection and classification of inertial signals to learn and recognize abnormal human movement. Our model was experimentally evaluated on four benchmark datasets: Intelligent Media Wearable Smart Home Activities (IM-WSHA), a self-annotated physical activities dataset, Wireless Sensor Data Mining (WISDM) with different sporting patterns from an IM-SB dataset and an SMotion dataset with different physical activities. Experimental results show that the proposed feature extraction strategy outperformed others, achieving an improved recognition accuracy of 81.92%, 95.37%, 90.17%, 94.58%, respectively, when IM-WSHA, WISDM, IM-SB and SMotion datasets were applied.

## 1. Introduction

Recent developments in the healthcare industry help patients, especially the elderly, to avoid illness, accidents and disease [1]. Such strategies have introduced monitoring devices such as wearable, vision and marker-based sensors that secure, examine and improve human life in uncertain situations [2,3] while patients remain mobile. Wearable technology has replaced traditional diagnostics by delivering ubiquitous access to vital patient data via smartphones and wearable sensory clothing [4,5]. Wearable devices provide real-time feedback from sensor fusion and they allow for the deployment, analysis and exploitation of the acquired data. Patients, carers and health practitioners can use data gleaned from wearable inertial sensors to keep up-to-date with the health and wellbeing status of their clients. Such data are functional for healthcare industries, where they can be used to improve the living standards of humans through remote monitoring [6,7,8,9] and by providing data for further research and development. Rapid growth in the number of healthcare applications has had a profound impact on the assessment and the evaluation of the fitness models proposed so far [10,11]. This growth has been driven by the development of the Internet and wearable technologies. However, wearable technologies face challenges for life-log monitoring because they lack contextual information. In addition, some limitations, such as unstable human body movements, hardware limitations and ergonomic measurements, adversely affect the precision of devices made for human life-log monitoring and recording [12].

Technological advances in wearable sensors have resulted in increased demand in research for security, healthcare and wellbeing applications such as security and surveillance systems, mental wellness apps, personal trainer assistants, smart homes and assistive robots [13,14,15,16,17,18,19]. In security and surveillance systems, applications that detect uncertain or abnormal events use physical activity monitoring systems that prompt precautionary measures against violent acts [20,21]. In mental wellness, apps help those concerned to make choices towards healthy, comfortable and safe lifestyles and behaviors by being sensitive to emotional and physical indicators. With regard to fitness training, wearable sensor technologies provide motion tracking in order to make training efficacious and efficient [22]. Truly smart home environments provide real-time physical monitoring of children and elderly people who need support due to developing, underdeveloped or deteriorating cognitive skills, respectively. The support offered by such devices enhances the wearer’s functional independence and quality of life. Furthermore, physical activity recognition systems deployed in homes help carers and family members to supervise and respond to elderly patients in a pervasive manner [23,24].

Recently, there has been a great demand for various applications for body-worn sensors. These revolutionary developments have impacted multiple aspects of human life, especially in healthcare and daily life monitoring. Among these wearable sensors, our work mainly focuses on inertial measurement unit (IMU) sensors such as accelerometers, gyroscopes and magnetometers, which enable us to examine human life in different routines and postures in order to detect changes in location, body movement and rotational changes in three-dimensional space [25,26,27]. In addition, healthcare industries make use of these sensors to monitor physiological and physical activities [28,29]. However, these sensors can also be used to sense sudden changes in the wearer’s posture or position, like falling, and this information can be used to help prevent falling and/or to dispatch prompt assistance, especially to the elderly [30,31]. Despite the feasibility of such wearable sensors, some challenges remain, such as the continuous monitoring of the data acquired from the sensors and the volume of data on the system. Such data are difficult to handle in real-time.

This paper mainly focuses on the optimization of healthcare physical activity recognition systems that are intended to reduce difficulties in monitoring human physical routines via IMU-based wearable sensors, which measure the movements, postures and orientations of those wearing the sensors. The proposed physical activity recognition system comprises four main steps: the placement of sensors, a signal denoising process, feature selection and data classification. Initially, we placed three inertial sensors (i.e., accelerometers, gyroscopes and magnetometers) at different body locations (i.e., chest, thigh and wrist). Acquired data were filtered with a third-order median filter to eliminate impulsive types of noise; this restored the signal to close to normal motion. Then, we adapted several approaches of statistical and non-statistical features. For sustained signal data, relevant descriptive features that contribute more to the recognition of human physical activities in unlike conditions were selected. Finally, a reweighted genetic algorithm model was embodied in the model to recognize and set parameters to classify human activities from feature vectors to attain significant accuracy. To evaluate performance, we applied our proposed model to the IM-WSHA dataset, which is based on diverse patterns of physical activities. Simultaneously, we employed the proposed model for two public benchmark datasets: the WISDM and IM-SB datasets. The major contributions of our paper are highlighted as follows:

- The fusion of multiple features from different domains makes the proposed physical healthcare detection system robust even with noisy data and holds the local dependent properties over the reweighted genetic algorithm acting as a novel methodology in order to improve the recognition rate over all three human activity datasets.
- For complex human physical activity patterns, we designed a novel genetic algorithm-based pattern matching method that provides contextual data coupled with classifying behaviors.
- Moreover, a comprehensive analysis was carried out on two public benchmark datasets (WISDM and IM-SB) along with a self-annotated dataset (IM-WSHA) for human physical healthcare activities; this achieved notable results compared with other state-of-the-art methods and deep learning algorithms.

The rest of the paper is organized as follows. Section 2 offers a brief overview of related work in the area of human healthcare activity analysis. Section 3 presents the proposed architecture of our human healthcare activity model. Section 4 comprises the details of one self-annotated dataset and two public benchmark datasets along with the experimental setup and results. Finally, Section 5 presents the conclusions and describes research perspectives.

## 2. Related Work 

### 2.1. Features-Based Healthcare Activity Recognition Systems Using 2D/3D Cameras

Image processing techniques have been used prolifically to recognize human movement patterns from 2D/3D video and still images. Advances in multimedia tools and sensing devices have made it easier for researchers to track and analyze human positions and postures by employing techniques like foreground segmentation, silhouette extraction, etc. For instance, Liu et al. [32] analyzed human activity recognition (HAR) for healthcare using RGB-Depth cameras. They extracted both 2D and 3D movements, still postures and transition actions. Nonlinear Support Vector Machine (SVM) is applied to classify different human activities. In [33], Crispim et al. proposed a multi-sensor surveillance system for older patients based on video cameras in order to automatically detect life events. The proposed system was tested with nine participants. Their multi-sensors approach shows an improvement over vision-based systems. Zouba et al. [34] utilized a cognitive vision approach to detect and identify daily living activities based on 3D representations of key human postures. They modeled multiple video events in real-time scenarios. However, this approach was only evaluated experimentally on small datasets.

In [35], Wu et al. presented a hierarchical approach to recognize multi-view activities in home environments with different visual features and learning methods. Their proposed method focused on different fusion techniques such as spatio-temporal features, decision methods and feature fusion methods for multi-view activity recognition. Kim et al. [36] analyzed a depth vision-based human activity recognition system for older people’s healthcare in home environments. Their model processed convolving features such as joint distance, magnitude and centroid features, which are used for feature extraction. Furthermore, for classification, they used a Hidden Markov Model (HMM) to recognize various human activities.

### 2.2. Features-Based Healthcare Activity Recognition System Exploiting Wearable Sensors 

Human motion analyses have been strengthened by recent advances in electronics, especially due to the introduction of Micro Electro-Mechanical Systems (MEMS). Micro versions of electronic sensors have added to comfort and adaptability to daily routine motion detection. In their efforts to design human motion instruments, researchers have employed a combination of sensors to find better solutions for analyzing skeletal movements and the quantization of human motion. MEMS sensors (accelerometers, gyroscopes and magnetometers) in particular have been playing a significant role in the recording and analysis of motion data [37]. In [38], Leonardis et al. focused on a multi-featured technique to recognize eight human activities with magnetic and inertial measurement unit (MIMU) sensors. They processed all signals from a tri-axial set of sensors: accelerometer, gyroscope and magnetometer. In addition to feature extraction, appropriate features were selected to maximize performance. Finally, state-of-the-art classifiers were applied to evaluate the benchmark performance. Zebin et al. [39] presented deep learning techniques for a human activity recognition system using body-worn inertial sensors. They presented feature learning methods for their activity recognition system. In addition, convolutional neural network (CNN) architecture is applied to automate feature learning for the recognition of different activities. In [40], Margarito et al. assessed the dynamics of human motion by placing an accelerometer sensor on the user’s wrist. They captured motion data representing eight healthcare patterns which were compared against existing pattern templates using Euclidean distance and dynamic time warping (DTW). In addition, statistical learning approaches were applied for the segregation of quantized motion data.

Xu et al. [41] proposed a novel method of activity recognition using wearable sensors by combining three IMUs and one heart rate sensor. These sensor streams were subjected to multi-feature extraction from Hilbert–Huang transform to enhance human activity recognition. In [42], Jalal et al. dealt with accelerometer signals statistically and applied a linear support machine to classify quantized human motion data produced from accelerometer sensors. Nweke et al. [43] conducted research to analyze human activity recognition and health monitoring using multi-sensor fusion, namely accelerometers and gyroscopes on two public benchmark datasets. The authors critically analyzed multiple techniques for data fusion, feature selection and classification techniques for human physical activity recognition via inertial-based sensors. 

## 3. Materials and Methods

### 3.1. Overview of the Solution Framework

**Figure 1:**
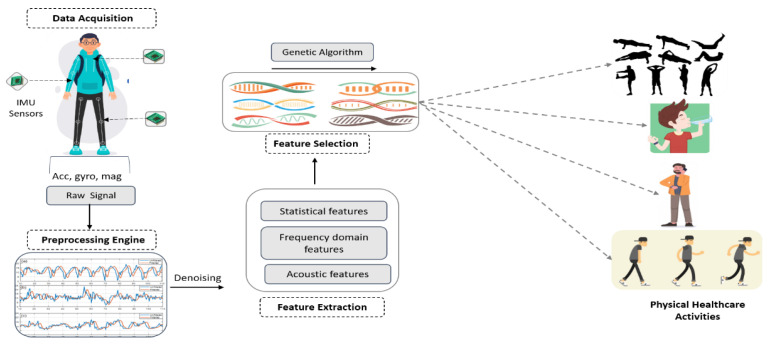
Flow architecture of the proposed physical healthcare detection system.

The proposed system recognizes the physical healthcare activities of humans via three inertial measurement units: accelerometer, gyroscope and magnetometer sensors. The proposed model’s architecture is elicited in Figure 1. The process is divided into four phases: signal preprocessing, feature extraction, feature selection evaluation and a genetic algorithm-based classifier. Initially, the sensor data are rectified by applying a filtering scheme to deal with the noisy peaks resulting from abrupt movements. These signals are further processed to compensate for delays introduced as a result of signal filtering. Secondly, the smoothened signal values are arranged into time-blocks of consistent duration for the extraction of signal features. In feature extraction, a set of descriptive statistical and non-statistical feature vectors are extracted such that the signal data are represented by minimal possible information. Moreover, the extracted features are normalized with the help of extremes to hinder any complex value from occurring in the later stages of feature evaluation and selection. The feature extraction phase is then followed by feature selection. Thirdly, feature selection is a compression technique applied to feature vectors such that the contributing features are maintained for the later stages of data evaluation. The contributive nature of a feature is defined by the threshold that is calculated as a mean of the previous evaluations. Lastly, the processed signal data and selected features are supplied to the classifier algorithm, which assesses the signal stream and applies only the compressed feature set for training and testing the model.

### 3.2. Signal Pre-Processing

**Figure 2:**
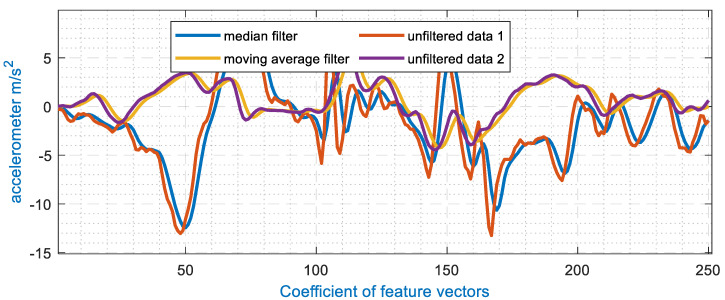
Signal preprocessing for wearable accelerometers in the proposed healthcare model.

For any system involving signal analysis, pre-processing is the key to maintaining the shape of the data. In the proposed model, accelerometer signals are processed by a moving filter to enhance the signal and smoothen extreme points. Moreover, these smoothened signal streams are normalized for the abduction of negative values from the system to avoid the occurrence of non-real values in the feature extraction phases. The processed and noisy signal components of the median and moving average filters of the accelerometer can be seen in Figure 2.

### 3.3. Feature Extraction

**Figure 3:**
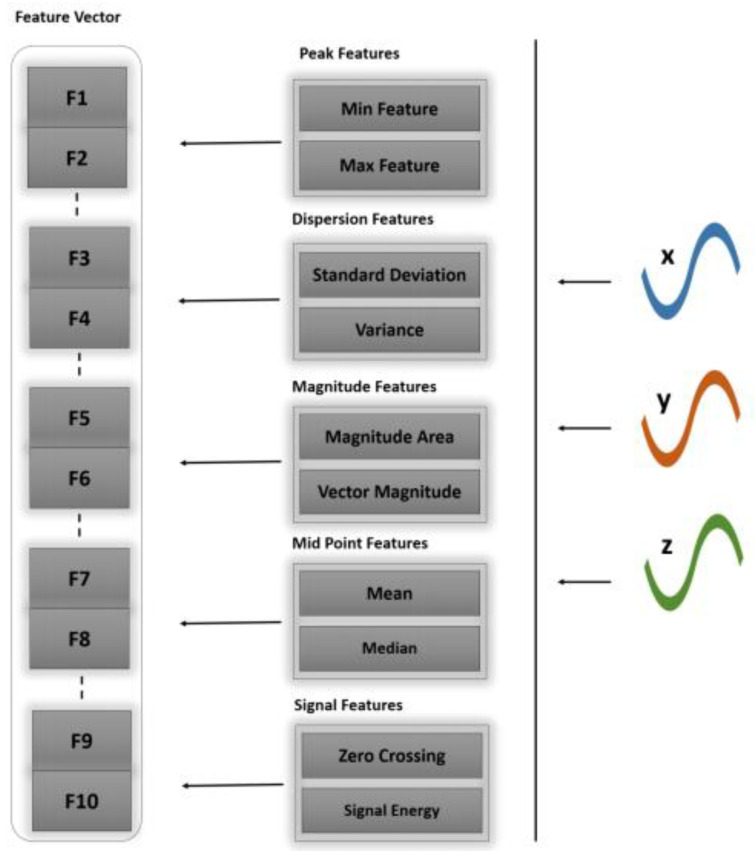
Features vectorized representation of inertial sensor stream components x, y, z.

Feature extraction is a prominent phase in all machine learning systems, where the emphasis lies on representing information with a meaningful set of attributes that covers the whole scenario. In our proposed model, statistical features have been used to facilitate the analysis of accelerometer signals. The denoised signals are taken as a stream and subjected to feature extraction for the sensor data stream. Initially, the definitive parameters are considered, namely window selection and signal overlap region [44]. Furthermore, signal attributes are extracted from within the bounding region with sufficient contextual information. Algorithm 1 explains the multi-fused feature extraction model. Figure 3 shows the vectorization of features with statistical features. 

In the first algorithm, we explained how the inertial raw signal (accelerometer, gyroscope and magnetometer) data are acquired. Then, we applied the third-order median filter technique to remove noise and restore the shape of the signal to near normal motions. Then, we adapted the sliding window approach, which consists of splitting the inertial-based sensor data into batches of equal size for the analysis of human motion patterns. Finally, we acquired the framed data to extract statistical, frequency and acoustic features. Then, we combined all the features into a vector/matrix for further processing.
**Algorithm 1: Multi-fused inertial signal (acc, gyro, mag) feature extraction****Input**: acc = acceleration data (x,y,z), gyro = gyroscope data (x,y,z), mag = magnetometer data (x,y,z), WS = window size and SR= sampling rate (100 Hz). **Output**: feature vector for physical healthcare activities (PHA). feature_vector ← [] window_dimension ← AcquireWindow_dimension ()/* acquire window size of inertial signal */ over_lap ← Acquirelap_time()      /* Get overlapping time */ **Method** PHA(IMU(acc,gyro,mag)) Multi-FusedVector <- []    /* Combine inertial signal data for preprocessing*/ Filtered_Data <- MovingAverageFilter(acc, gyro, mag) /*acquire frame data from filtered data(sampled and windowed) */ Frame_Data(Filtered_Data, SR, WS) **While** exit condition not true **do** /* Extracting statistical, frequency, and acoustic features */ statistical_features <- ExtractStatisticalFeatures(Frame_Data)/* extract statistical features */ frequency_features <- ExtractFrequencyFeatures(Frame_Data)/*extract frequency-based features */ Acoustic_features <- ExtractAcousticFeatures(Frame_Data)/* extract acoustic features */ /* appending all above calculated features into one vector */ Multi-FusedVector <- [statistical_features, frequency_features, acoustic_features] **end while** **return** Multi-FusedVector

#### 3.3.1. The Signal Magnitude Feature

Regarding the signal magnitude feature Sig(mag), we measure the distance between actual points *i* of the coordinate signal points within the period to perceived different activities as

(1) $$\mathrm{Sig}\left(\mathrm{mag}\right)=\sqrt{x_{i}^{2}+y_{i}^{2}+z_{i}^{2}}$$

where *x(i)* is the actual point value of signal *x*, for *y(i)* and *z(i)* of each windowing signal.

#### 3.3.2. The Zero Crossing Rate Feature

**Figure 4:**
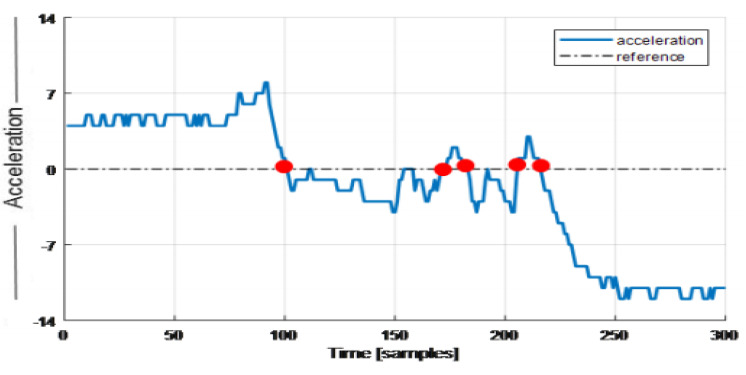
The instantaneous vector magnitude for the walking signal pattern.

The zero crossing rate (ZCR) is the measure of a signal interchange having an amplitude from the negative to the positive region and vice versa. A count of zero crossing rate gives a good insight into the signal variation with respect to changing time. Significantly, ZCR is used to measure the measuring pitch of our inertial signal analysis, as shown in Figure 4.

#### 3.3.3. Peak Features

The peak signal feature Sig(min_e_) is extracted from triaxial components by measuring the actual acceleration component in order to find the minimum and maximum values in the respective sequences of signals:

(2) $${\mathrm{Sig}\;(\min}_{e}{)\;=\;\min\;(e\;(p\;<\;Q}_{s})\;)$$

(3) $${\mathrm{Sig}\;(\max}_{e}{)\;=\;\max\;(e\;(p\;<\;Q}_{s})\;)$$

where e represents the signal types, i.e., x, y, z of the acceleration signal, p is the current value, and Q_s_ provides quartile values having negative peak.

#### 3.3.4. Standard Deviation Feature

**Figure 5:**
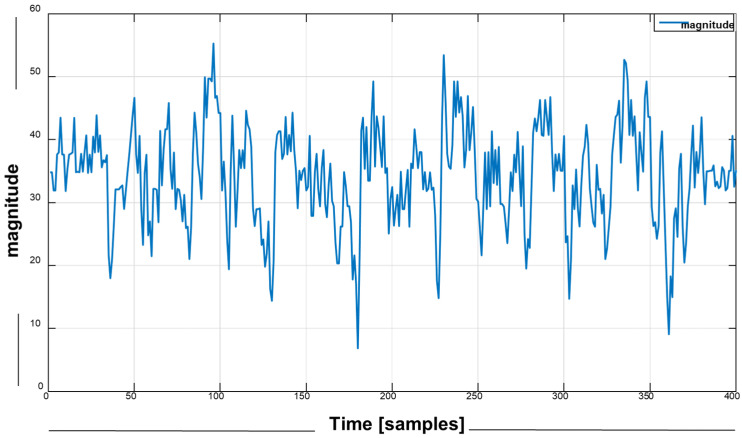
The instantaneous vector magnitude for the walking signal pattern from accelerometer sensor.

In the standard deviation feature, we measure the possible deviation of acceleration signals and the mean value from respective sequences of signals. Thus, the sequence obtained as a result of possible deviation is given as

(4) $$\mathrm{Sig}\left(\mathrm{std}\right)=\sqrt{\sum_{i=1}^{n}{\left(X_{i}-\bar{X}\right)}^{2}/n-1}$$

where X_i_ is the value of the processed signal. In Figure 5, the varying acceleration is presented against the mean having dispersion around the total mean. Thus, the closer the data around the mean, the more likely the chance of obtaining the standard deviation as a good predictor.

#### 3.3.5. Magnitude Area Feature

The signal magnitude area, which is calculated according to Equation (5), is used to derive a measure of the subject’s level of activity. It can distinguish between periods of acceleration and non-acceleration (static periods) thus:

(5) $$\mathrm{Area}\left(\mathrm{mag}\right)=\sum_{i=1}^{n}X_{i}+\sum_{i=1}^{n}Y_{i}+\sum_{i=1}^{n}Z_{i}$$

where X_i_, Y_i_, and Z_i_ indicate the acceleration signal along with the *x*-axis, *y*-axis, and *z*-axis, respectively. 

#### 3.3.6. Mean Feature

**Figure 6:**
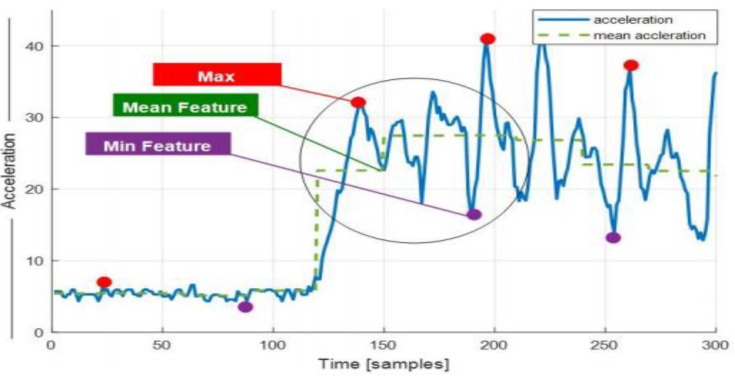
The three statistical features (mean, min, max) for practicing imitation in the “climbing upstairs” motion pattern using the Wireless Sensor Data Mining (WISDM) dataset.

The mean is a statistical feature and an important ingredient in many other features, providing an intuition into the signal’s overall energy over the course of time. Features like standard deviation, variance and zero crossing are totally reliant on the mean for the calculation of these features. Figure 6 represents a 1D plot with a fusion of different statistical features of the ascending motion pattern using the WISDM dataset. 

#### 3.3.7. Spectral Entropy

**Figure 7:**
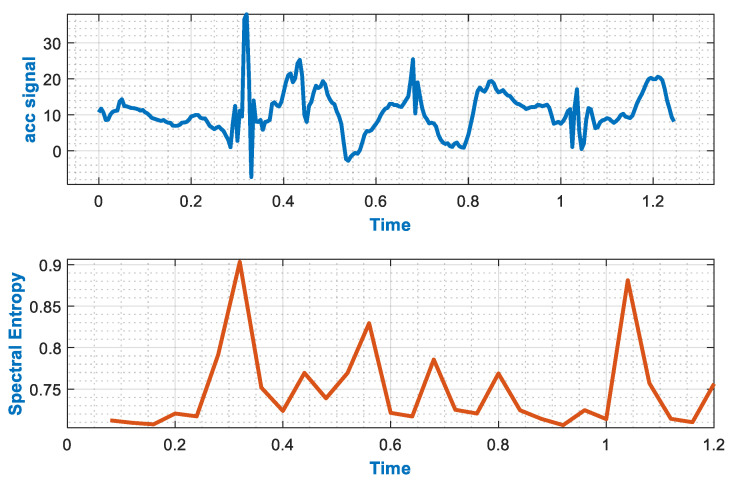
Spectral Entropy for brushing hair signal pattern using the Intelligent Media-Wearable Smart Home Activities (IM-WSHA) dataset.

Spectral entropy is used as a feature to describe the complexity of a system. This complexity of the system provides vital information which helps to determine the spectrum of inertial signals [45]. It also helps to determine the Power Spectral Density of inertial signals. In addition, spectral entropy handles the normalized power distribution of that signal in the frequency domain and determines its Shannon entropy. This feature is useful in finding any uncertain peaks, e.g., sudden falls that occur during normal motion. Spectral entropy of the inertial signal for the frequency band $f_{1}-f_{2}$ is represented thus:

(6) $$S_{N}(f_{1},f_{2})\;=\;\frac{-\sum_{f_{i}=f_{1}}^{f_{2}}\;\;P(f_{i})\log\;(P(f_{i}))}{\log\;(N[f_{1},f_{2}])}$$

where $P\;(f_{i})$ shows the value of frequency $f_{i}$, in Power Spectral Density. Furthermore, $N[f_{1}{\text{-}f}_{2}]$ is the number of frequency components in the denoting band during Power Spectral Density determination. Figure 7 visualizes the spectral entropy for the hair brushing motion pattern using the IM-WSHA dataset.

#### 3.3.8. Hilbert–Huang Transform (HHT)

**Figure 8:**
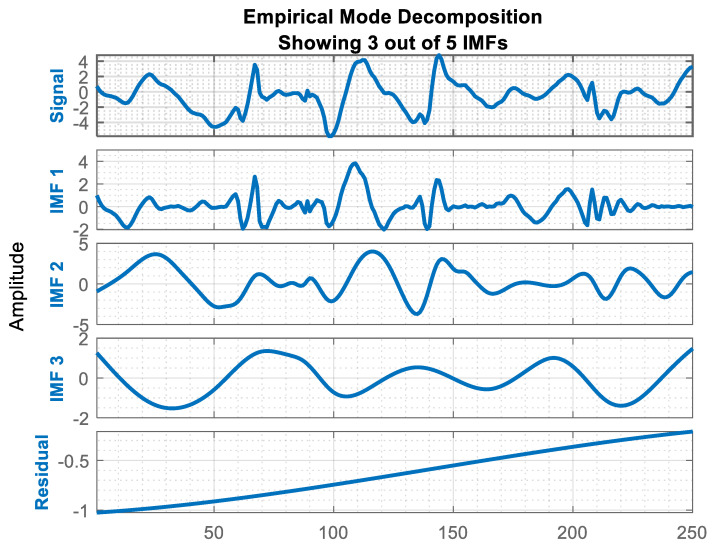
The empirical mode decomposition components from the inertial data.

Hilbert–Huang transform (HHT) is useful for dealing with varied signal data [46]. In this paper, we employ HHT to analyze inertial signals to deal with variable patterns. Hilbert–Huang transform involves the Hilbert transform and Empirical Mode Decomposition (EMD). EMD plays a vital role in our inertial data. It decomposes the inertial data into a fixed and small number of IMFs called intrinsic mode functions [47]. These IMFs are used to extract features and they are pooled with time-domain values to analyze some statistical patterns. Finally, all these features are used to create a combined feature vector. Figure 8 represents the intrinsic mode function from the inertial data.

### 3.4. Feature Selection

**Figure 9:**
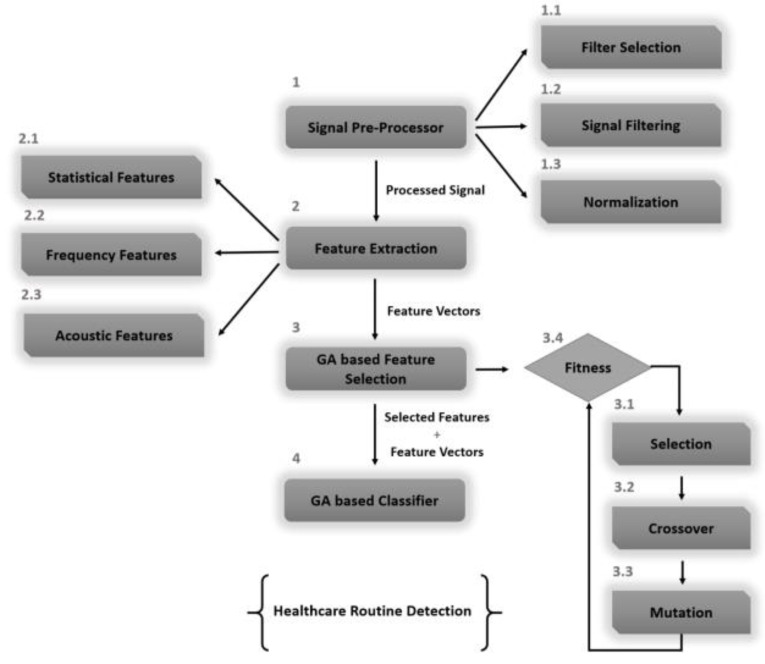
The proposed reweighted genetic algorithm for recognizing human motion from inertial-based data.

Due to the high computational cost associated with signal processing, especially when it includes contextual information, the importance of feature selection cannot be underestimated. Genetic algorithm (GA) is an evolutionary strategy that follows the principles of natural selection for the evolution of the next generation. The proposed algorithm uses GA to find a set of features that embrace all the significant information nodes from the inertial signals (see Figure 9). In the assimilation of inter-signal variation(s), the process is led by biological crossover and mutations to bring stochasticity to the process. In addition, features are assigned random weights to help model non-linear behavior in understanding complex signal patterns. Crossover operation is governed by the principle of producing offspring from a set of selected parents. Furthermore, randomization is applied to feature vectors for the fulfillment of mutation operations. Mutation plays a significant role in fast convergence of the algorithm but often leads to a reduced computational cost. Feature selection is an important phenomenon that keeps the population pool filled with a mix of the fittest average feature sets. Algorithm 2 represents the genetic algorithm based on a reweighted feature selection method.

(7) $$\sigma \left(X,Y,Z)=\sum_{i=1}^{n}\sum_{j=1}^{w}(X_{\mathrm{time}},X_{\mathrm{freq}},X_{\mathrm{acoustic}}\cdots Y_{\mathrm{time}},Y_{\mathrm{freq}},Y_{\mathrm{acoustic}}\cdots Z_{\mathrm{time}},Z_{\mathrm{freq}},Z_{\mathrm{acoustic}}\right)$$

Equation (7) comprises the feature extraction stage for the inertial signal data, where X_(time, freq, acoustic)_, Y_(time, freq, acoustic)_ and Z_(time, freq, acoustic)_ embody signal streams. Each signal segment is employed to extract the time, frequency and acoustic features. 

In the second algorithm, we explained how we acquired the multi-fused feature from a vector. The feature vector is then converted into corresponding chromosomes. Then, these multi-fused features are further processed and reweighted to extract an optimal weight. After extracting optimal weights, we need to calculate crossover chromosomes and global maxima. Finally, we obtain relevant features based on Linear Support Vector Machine (LSVM) and the random forest-based fitness function.
**Algorithm 2: Genetic algorithm-based reweighted feature selection****Input**: FV: Multi-fused feature vectors (u_1_, u_2_, u_3_,… u_n_) /* acquire feature vector */ **Output**: FL: Multi-fused feature list (l_1_, l_2_, l_m_) /* obtain vector of optimal features*/ /* feature vectors are converted into corresponding chromosomes */ **for** vector in population_lab_
**do** /* multi-fused-feature vectors are further processed and reweighted to extract an optimal weight  RewightedFeatures <- [] **while** fitness not achieved or fitness not changing **do**  **for** feature in vector **do**   **ReweightedFeatures (feature)**  **end for**  **Rechoose ()**  offspring1, offspring2 <- CrossOver (vector) /* calculate crossover chromosomes*/  mutated <- Mutation (vector)     /* calculate global maxima */ /*obtain relevant features on the basis of Linear Support Vector Machine (LSVM) and random forest-based fitness function*/  Evaluationfunction <- GetFitness (vector) **end while** **return** ReweightedFeatures **end for**


In Equation (8), the symbol χ represents the process of applying a crossover between two-parent chromosomes $C,\bar{C}$ in the feature vector. In Equation (9), the χ illustrates the addition of mutations in crossed children to deal with the same chromosomes. All chromosomes are fixed versions of features to represent each bit as a trait.

(8) $$\chi \left(C,\bar{C}\right)=X_{c1}..X_{cn},Y_{c1}..Y_{cn},Z_{c1}..Z_{cn}{\overset{\times }{X}}_{c1}.{\bar{X}}_{cn},{\bar{Y}}_{c1}.{\bar{Y}}_{cn},{\bar{Z}}_{c1}.{\bar{Z}}_{cn}$$

(9) $$C\prime =\chi \left(C,\bar{C}\right)=X\prime_{c1}..X\prime_{cn},Y\prime_{c1}..Y\prime_{cn},Z\prime_{c1}..Z\prime_{cn}$$

In order to avoid replication of uniform chromosomes in the population, mutation is introduced to the crossed children also shown in Equation (9). At the start, the mutation rate is set to 0.05 to avoid primitive randomization. In this way, mutation maintains the divergence by adapting another level of randomization with the new generations. In Equation (10), mutations are represented by *μ*($F^{\prime }$).

(10) $$\mu \left(F\prime \right)=\{\begin{array}{c} X\prime_{C1}\ldots X\prime_{C_{n}},Y\prime_{C1}\ldots Y\prime_{Cn},Z\prime_{C1}\ldots Z\prime_{Cn}\\ Y\prime_{C1}\ldots {\hat{Y}}_{Cn},Z\prime_{C1}\ldots Z\prime_{Cn},X\prime_{C1}\ldots {\dot{X}}_{Cn} \end{array}$$

Finally, we introduce the reweighted genetic algorithm [48], which consists in giving weights to specific features while avoiding others. In this way, we did not need to try all possible combinations of weights, which would increase computation with the conventional genetic algorithm. The weight assignment in GA strengthens the selection and classification process as an output. In Equation (11), $W_{a1}$ are defined as weights and a1 is the feature depicted in the chromosomal structure.

(11) $$RGA\;(C\prime )=\;W_{a1}.Y\prime_{c1}\ldots W_{an}.{\hat{Y}}_{cn},W_{b1}\cdot Z\prime_{c1}\ldots W_{bn}\cdot {\hat{Z}}_{cn},W_{c1}\cdot X\prime_{c1}\ldots W_{cn}\cdot X\prime_{cn}$$

where $\vartheta {\left(\dot{C}\right)}_{\mathrm{lsvm}}$ accounts for the classification result of the support vector machine and $\vartheta {\left(\dot{C}\right)}_{\mathrm{rf}}$ are utilized for the random forest accuracy results.

(12) $$\mathrm{Fitness}\left(C^{\prime }\right)=\frac{\vartheta {\left(C\prime \right)}_{svm}+\vartheta {\left(C\prime \right)}_{rf}}{2}$$

### 3.5. Genetic-Based Classifier

**Figure 10:**
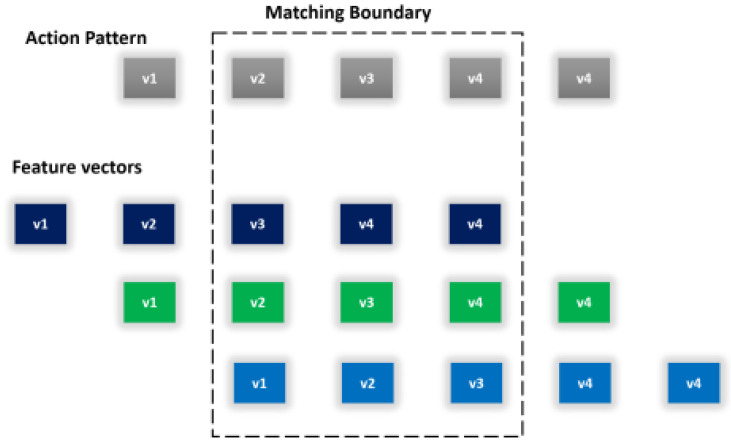
The reweighted pattern-matching algorithm for human physical healthcare pattern understanding.

Classification refers to the taxonomic grouping of data into respective groups based on similarity. Categorization is achieved by drawing clear-cut boundaries between the classification groups. The genetic algorithm’s evolutionary nature has been used to evolve discriminating separators between different classes by exploiting the differences in feature vectors. In the proposed model, GA has been used to solve complex pattern-matching problems. Motion data require closely related signal patterns that can cope with inter-class similarity. Following the same steps involved in feature selection, the genetic algorithm uses the biological operations of crossover and mutation to shuffle feature vectors until the maximum possible boundary is marked between the classes. Figure 10 shows the reweighted pattern-matching algorithm for human physical healthcare pattern understanding. In Equation (13), we classified the labeled behaviors in inertial signal patterns with high similarity ratio comprising its context as

(13) $$\mathrm{Fitness}(p^{\prime })\;=\;.({p^{\prime }}_{1}{p^{\prime }}_{2}{p^{\prime }}_{3}{p^{\prime }}_{4}{p^{\prime }}_{5}{p^{\prime }}_{6}{p^{\prime }}_{7}{p^{\prime }}_{8}{p^{\prime }}_{9})\%(\begin{array}{c} p_{11}p_{12}p_{13}p_{14}p_{15}p_{16}p_{17}p_{18}p_{19}\\ p_{21}p_{22}p_{23}p_{24}p_{25}p_{26}p_{27}p_{28}p_{29}\\ p_{31}p_{32}p_{33}p_{34}p_{35}p_{36}p_{37}p_{38}p_{39}\\ p_{41}p_{42}p_{43}p_{44}p_{45}p_{46}p_{47}p_{48}p_{49}\\ p_{51}p_{52}p_{53}p_{54}p_{55}p_{56}p_{57}p_{58}p_{59}\\ p_{61}p_{62}p_{63}p_{64}p_{65}p_{66}p_{67}p_{68}p_{69}\\ p_{71}p_{72}p_{73}p_{74}p_{75}p_{76}p_{77}p_{78}p_{79}\\ p_{81}p_{82}p_{83}p_{84}p_{85}p_{86}p_{87}p_{88}p_{89}\\ p_{91}p_{92}p_{93}p_{94}p_{95}p_{96}p_{97}p_{98}p_{99} \end{array})$$

## 4. Experimental Results

The proposed system is evaluated by a genetic algorithm-based classifier. It includes one self-annotated Intelligent Media Wearable Smart Home Activities (IM-WSHA) dataset and two public benchmark datasets named WISDM and IM-SB, respectively. These datasets include multiple physical healthcare activities in different indoor/outdoor environments, i.e., smart home, sports ground and public places.

### 4.1. Intelligent Media Wearable Smart Home Activities Dataset (IM-WSHA)

The Intelligent Media Wearable Smart Home Activities [49] is our self-annotated dataset, which comprises three wearable IMU sensors (MPU-9250). This dataset contains 220 sequences of accelerometer, gyroscope and magnetometer data. These sensors were positioned at the wrist, chest and thigh regions to capture different aspects of human body motion. Ten participants (five males and five females) performed 11 different physical healthcare activities in smart home environments, namely phone conversation, vacuum cleaning, watching TV, using computers, reading books, ironing, walking, exercise, cooking, drinking and brushing hair. The participants involved included both young and old people whose ages ranged between 19 and 60 and whose weight ranged between 55 and 85 kg. The usage of multisensory devices adds challenges when dealing with rigorous motion data [49].

The reweighted genetic algorithm was tested on an IM-WSHA dataset to analyze physical healthcare activity data from different dimensions and was compared with a conventional linear support vector machine (LSVM). Table 1 illustrates the performance matrix of human activity recognition for 11 different activities with a mean accuracy of 81.92%. 

To determine the optimal parameters for a reweighted genetic algorithm to function properly, different sliding ratios were accommodated to find the perfect balance between the sliding ratio and the accuracy of the proposed system. It is worth noting that the sliding ratio accounts for the contextual information needed for the later part of the signals. The application of contextual information is a prominent factor which holds the sequence of events as a single chain and allows a better understanding of motion patterns in context. Thus, Table 2 depicts the impact of different sliding ratios for the IM-WSHA dataset. It is clearly observed that the proposed approach resulted in average results, especially for non-repetitive activities like phone conversations, watching TV, using computers, reading books and cooking. These five physical activities are movements without repetition, which causes lower accuracy compared to other activities. On the other hand, activities such as walking, exercise, vacuum cleaning, ironing, brushing hair and drinking movements, with repetition in terms of the subject’s body movements, produce high accuracy rates.

In Table 3, we tested different state-of-the-art methods using our IM_WSHA dataset. In [50], we extracted statistical features from our dataset and then classified the physical activities with multilayer feedforward neural networks and achieved 73.27% accuracy. In [51], we applied decision trees to our dataset and achieved 78.19% recognition accuracy. In Attal et al. [52], we extracted both time and frequency domain features fused with the Hidden Markov Model (HMM) on our IM-WSHA dataset and we achieved 80.37% recognition accuracy. Finally, we applied the proposed model to our self-annotated dataset and achieved significant recognition accuracy of 81.92%. 

### 4.2. WISDM Dataset

The Wireless Sensor Data Mining (WISDM) [54] dataset is a large repository of smartphone-based motion data which involves transient motion data. The dataset accommodates routine motion patterns with a significant number of processable motion samples. The daily life routines possessed by the dataset could possibly be used to analyze the movement of the different body components of the elderly. In the analysis of body positioning for the very elderly, the postural positioning and coherence between body parts can be achieved by recording the quantized motion. With the usage of built-in smartphone sensors, the subject’s movements are translated into acceleration signals. These signals can be used for the identification of a subject’s movements in daily life routines. The WISDM dataset involves six main motion patterns, i.e., walking, jogging, ascending, descending, sitting and standing. For a balanced ratio of sensor data, a sampling frequency of 50ms was used to stream acceleration signals.

The proposed reweighted genetic algorithm was applied to the WISDM dataset to analyze the performance of the proposed dataset. Moreover, a linear support vector machine (LSVM) and random forest were used as second and third classifiers to check physical activity performance, as shown in Table 4.

As Table 5 shows, 10%, 30% and 60% sliding windows were applied to the signal streams and corresponding results were produced. The 10% sliding window produced only average results in terms of accuracy because less consideration was given to the context, but the 30% and 60% sliding windows produced significantly higher results. Therefore, our proposed strategy adopted a 60% overlapping sliding window.

Similarly, the classification results not only reveal the non-contributive features, but they also assign weights to the prominent contributive features. With the usage of weights, non-linearity is introduced into the model, bringing more flexibility to our understanding of motion patterns that involve a high level of variability in terms of axial signals. In the assessment of weights assigned to different features, the results of some trials are presented in Table 6; these show the weighted values for each feature according to the trials performed. Here, Table 6 shows the correlation feature that contributes far less than any other feature. 

The performance results of the proposed work are compared in Table 7, where the reweighted genetic algorithm excels beyond other state-of-the-art models. These algorithm models involved learning systems as well as convolutional neural networks. However, in terms of dealing with variability, our proposed system results are slightly better than those of state-of-the-art models.

### 4.3. IM-SB Dataset

The Intelligent Media Sporting [58] dataset is a multisensory accelerometer-based dataset. The IM-SB dataset involves quantized motion data for physical sporting activities, i.e., badminton, basketball, cycling, football, skipping and table tennis. The dataset involves accelerometer sensors attached to the wrist, thigh and back of the subject to analyze movement from different dimensions. In Table 7, the reweighted genetic algorithm was tested on an IM-SB dataset to analyze sporting motion data from different dimensions and was compared with conventional LSVM. 

The controlling parameters for the reweighted genetic algorithm were also modified for the IM-SB dataset in order to check its performance. Again, the proposed accuracy applying the 60% sliding ratio suggested the adoption of a slightly bigger sliding window. Moreover, 10% and 30% contextual information failed to identify the movement from the later part of the signals. Table 8 shows the impact of varying sliding ratios for the IM-SB dataset.

In the pursuit of better parameters, the algorithm was run on several occasions to find the optimum measure of weights assigned to each attribute. Table 9 shows the reweighted values of the features in different trials.

Table 10 shows results for tests on different state-of-the-art methods against the IM-SB dataset. In [59], we classified six sporting behaviors with multiclass AdaBoost and achieved 73.67% accuracy. In Politi et al. [60], we extracted statistical and physical features from the IM-SB dataset and classified it using support vector machine (SVM), which achieved 78.41% recognition accuracy. In [61], we extracted statistical features fused with Multilayer Perceptron (MLP) from an IM-SB dataset and we achieved 87.38% recognition accuracy. Finally, we applied the proposed model to the IM-SB dataset and achieved a significant recognition accuracy rate of 90.17%. The performance results of the proposed work are compared in Table 11.

### 4.4. The Wearable Inertial Measurement (SMotion) Dataset

The Wearable Inertial Measurement Sensors (SMotion) [62] dataset is an inertial (SHIMMER3) based dataset. The dataset involves a SHIMMER device attached to the waist of the subject to capture devised motion patterns from different aspects of the body. These sensors are positioned at the wrist to capture different dynamics of human body motion. In total, 114 healthy subjects performed three different daily physical activities, i.e., walking, standing still and sitting down and getting up (out of a chair). The performance results of the SMotion dataset are compared in Table 12.

The reweighted genetic algorithm was tested on our four datasets to analyze physical healthcare activities from different dimensions and was compared with a conventional linear support vector machine (LSVM) and random forest, which achieved 94.58%, 91.45% and 94.58% accuracy, respectively.

Table 13 shows that 10%, 30% and 60% sliding windows were applied to the inertial signal streams and presents the corresponding results.

## 5. Conclusions

In this paper, we have reported the development of a robust approach that can precisely report the physical health and wellbeing status in four challenging benchmark datasets in both indoor and outdoor environments. In addition, we developed a novel framework which is comprised of statistical features, frequency features, transform and acoustic features to extract optimal features to detect and recognize human physical health and wellbeing via a triaxial set of inertial signals: accelerometer, gyroscope and magnetometer.

Furthermore, we presented a robust reweighted genetic algorithm that gives the variation of genetic information and fusion of windowed signal patterns, which helps us to understand random human physical activities that may relate to the subject’s health and wellbeing status. Our system includes data analysis, monitoring and signal inertial measurements as well as efficient feature extraction algorithms which can potentially outperform the recognition accuracy rates of other systems. The proposed system provides remarkable results compared to state-of-the-art systems.

In future work, we will further enhance the efficiency of our features by adding angular and displacement information in order to classify more complex daily physical healthcare activities, especially for older and impaired people.

## Figures and Tables

**Table 1:** IM-WSHA dataset applied over the proposed classifier and over well-known statistical classifiers.

Symbols	Activities	LSVM (%)	Random Forest (%)	Proposed (%)
P1	Phone conversation	68.34	70.03	73.67
P2	Vacuum cleaning	74.36	78.37	84.78
P3	Watching TV	66.08	69.84	72.43
P4	Using computers	69.34	72.64	74.57
P5	Reading books	70.13	73.89	77.18
P6	Ironing	84.43	88.43	91.24
P7	Walking	86.71	89.41	93.16
P8	Exercise	83.76	87.65	89.78
P9	Cooking	67.48	69.36	75.83
P10	Drinking	75.57	80.14	86.23
P11	Brushing hair	73.87	78.2	82.29
**Mean Recognition Accuracy**		74.55	77.99	**81.92**

Bold letters for Mean Recognition Accuracy of IM-WSHA dataset.

**Table 2:** IM-WSHA dataset results for different sliding windows.

Symbols	10% Slide	30% Slide	60% Slide
P1	52.18	63.58	73.67
P2	65.28	77.36	84.78
P3	56.38	66.08	72.43
P4	58.25	67.34	74.57
P5	63.76	71.13	77.18
P6	68.92	79.80	91.24
P7	72.58	84.71	93.16
P8	70.49	81.67	89.78
P9	59.29	69.41	75.83
P10	69.77	80.12	86.23
P11	63.86	75.42	82.29
Mean Recognition Accuracy	63.70%	74.23%	81.92%

**Table 3:** Physical activity recognition accuracy comparison of the proposed method with other state-of-the-art methods over IM-WSHA inertial data.

Methods	Algorithm Details	Recognition Accuracy of IM-WSHA
Yang et al. [51]	Statistical features fused with multilayer feedforward neural networks	73.27%
Bonomi et al. [52]	Classification with decision trees	78.19%
Attal et al. [53]	Time and frequency domain features wrapped with Hidden Markov Model (HMM) classifier	80.37%
**Proposed Work**	Statistical, transform, acoustic and frequency features fused with reweighted genetic algorithm	**81.92%**

Bold letters for Proposed Recognition Accuracy.

**Table 4:** WISDM dataset results over the proposed classifier and other well-known statistical classifiers.

Symbols	Activities	LSVM (%)	Random Forest (%)	Proposed (%)
W1	Walking	91.42	93.64	98.81
W2	Jogging	92.27	94.12	98.47
W3	Ascending	89.14	90.83	91.23
W4	Descending	90.32	91.64	92.07
W5	Sitting	92.18	92.27	93.18
W6	Standing	93.06	94.18	98.47
**Mean Recognition Accuracy**		**91.39**	**92.78**	**95.37**

Bold letters for Mean Recognition Accuracy of WISDM dataset.

**Table 5:** WISDM dataset results for different sliding windows.

Symbols	10% Slide	30% Slide	60% Slide
**W1**	71.41	83.31	98.81
**W2**	73.68	93.45	98.47
**W3**	81.23	95.56	91.23
**W4**	56.43	73.28	92.07
**W5**	59.55	87.65	93.18
**W6**	75.23	82.94	98.47
Mean Recognition Accuracy	69.58%	86.03%	95.37%

**Table 6:** Feature weights for different trails in the WISDM dataset.

Feature Type	Trail 1	Trail 2	Trail 3	Trail 4	Trail 5
Zero Crossing Rate	0.81	0.75	0.69	0.90	0.85
Fundamental Frequency	0.78	0.95	0.88	0.74	0.39
Signal Magnitude Area	0.58	0.52	0.71	0.61	0.57
Signal Energy	0.66	0.68	0.63	0.61	0.68
Mean	0.78	0.69	0.75	0.74	0.76
Median	0.66	0.54	0.69	0.68	0.71
Mode	0.23	0.18	0.40	0.16	0.18
Standard Deviation	0.10	0.08	0.94	0.01	0.29
Variance	0.89	0.92	0.01	0.85	0.79
Phase Angle	0.48	0.40	0.39	0.38	0.41
Correlation	0.10	0	0.05	0	0.06
Min	0.76	0.69	0.69	0.57	0.60
Max	0.61	0.62	0.60	0.69	0.60

**Table 7:** Comparison results of the proposed method over other state-of-the-art methods using the WISDM dataset.

Methods	Accuracy (%)
Star with learning [55]	71.20
Impersonal Smartphone-based Activity Recognition (ISAR) [56]	75.21
Ignatov’s Convolutional Neural Network (CNN) [57]	93.32
**Proposed Work**	**95.37**

Bold letters for Proposed Method Recognition Accuracy of WISDM dataset.

**Table 8:** The Intelligent Media-Sporting Behavior (IM-SB) dataset results over the proposed classifier and other well-known statistical classifiers.

Symbols	Activities	LSVM (%)	Random Forest (%)	Proposed (%)
S1	Badminton	68.16	77.83	84.21
S2	Basketball	70.11	80.78	87.19
S3	Cycling	95.13	84.45	93.26
S4	Football	78.35	81.87	86.69
S5	Skipping	87.16	86.14	94.43
S6	Table Tennis	91.11	89.48	95.24
**Mean Recognition Accuracy**		**81.67**	**83.42**	**90.17**

Bold letters for Mean Recognition Accuracy of IM-SB dataset.

**Table 9:** IM-SB dataset results for different sliding windows ratios.

Symbol	10% Slide	30% Slide	60% Slide
S1	30.29	34.23	84.21
S2	34.26	68.19	87.19
S3	44.18	78.24	93.26
S4	36.10	70.92	86.69
S5	78.26	80.13	94.43
S6	70.22	81.11	95.24
Mean Recognition Accuracy	48.88%	68.80%	90.17%

**Table 10:** Feature weights for different trials with the IM-SB dataset.

Feature Type	Trail 1	Trail 2	Trail 3	Trail 4	Trail 5
Zero Crossing Rate	0.64	0.72	0.81	0.90	0.85
Fundamental Frequency	0.71	0.79	0.76	0.74	0.46
Signal Magnitude Area	0.81	0.76	0.56	0.61	0.58
Signal Energy	0.34	0.54	0.90	0.61	0.68
Mean	0.76	0.86	0.81	0.74	0.24
Median	0.55	0.49	0.67	0.68	0.87
Mode	0.36	0.13	0.45	0.16	0.18
Standard Deviation	0.22	0.05	0.94	0.01	0.39
Variance	0.78	0.23	0.08	0.85	0.68
Phase Angle	0.50	0.51	0.39	0.38	0.41
Correlation	0.05	0.02	0.05	0.02	0.06
Min	0.67	0.71	0.69	0.53	0.55
Max	0.58	0.24	0.60	0.54	0.72

**Table 11:** Comparison of the proposed method with other methods using the IM-SB dataset.

Methods	Algorithm Details	Recognition Accuracy of IM-SB Dataset (%)
Reiss et al. [59]	Classification with multiclass AdaBoost	73.67
Politi et al. [60]	Statistical and physical features fused with SVM	78.41
Yin et al. [61]	Statistical features wrapped with Multilayer Perceptron (MLP)	87.38
**Proposed Work**	Statistical, transform and frequency features fused with reweighted genetic algorithm	**90.17**

Bold letters for Proposed Method Recognition Accuracy of IM-SB dataset.

**Table 12:** The SMotion dataset applied over the proposed classifier and other well-known statistical classifiers.

Symbols	Activities	LSVM (%)	Random Forest (%)	Proposed (%)
L1	Standing	91.83	93.65	94.84
L2	Sitting down and getting up from chair	90.34	92.18	93.77
L3	Walking	92.18	94.63	95.13
**Mean Recognition Accuracy**		**91.45%**	**93.48%**	**94.58**

Bold letters for Mean Recognition Accuracy of SMotion dataset.

**Table 13:** SMotion dataset results for different sliding windows.

Symbols	10% Slide	30% Slide	60% Slide
L1	78.42	84.79	94.84
L2	81.36	86.67	93.77
L4	83.47	88.92	95.13
Mean Recognition Accuracy	63.70%	74.23%	94.58%
